# Supplementary material for: Netcombin: An algorithm for constructing optimal phylogenetic network from rooted triplets
Source: PLoS One. 2020 Sep 18;15(9):e0227842. doi: 10.1371/journal.pone.0227842 (PMC7500971; doi:10.1371/journal.pone.0227842)
Supplement: S4 File — (ZIP) [file pone.0227842.s004.zip › SequenceData-Triplet/sequenceData-10-20-30-0-Triplet/length 300, numseq 30, rate 10^-7/3 seq.pdf]

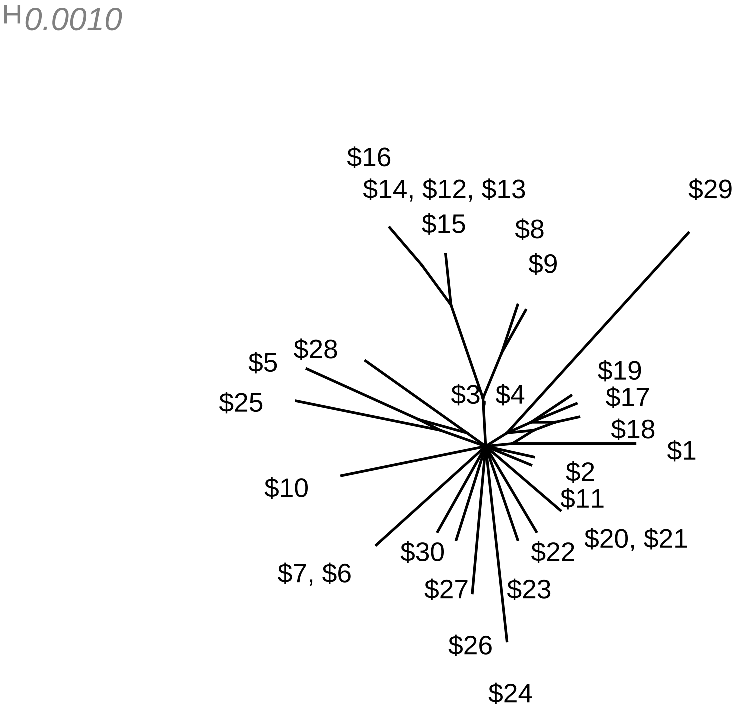

0.0010
$16
$14, $12, $13
$15
$29
$8
$9
$28
$5
$25
$3, $4
$19
$17
$18
$1
$2
$11
$10
$20, $21
$30
$22
$7, $6
$27
$23
$26
$24
